# Beyond *P_50_*, a better way to predict tree mortality

**DOI:** 10.1093/plphys/kiag204

**Published:** 2026-04-13

**Authors:** Alice Gauthey

**Affiliations:** Assistant Features Editor, Plant Physiology, American Society of Plant Biologists; Birmingham Institute of Forest Research, University of Birmingham, Edgbaston B15 2TT, United Kingdom

Drought-induced hydraulic failure is one of the main drivers of tree mortality globally (Choat et al. 2012). In a nutshell, the water-conducting system, the *xylem*, is under tension (or negative pressure, called *water potential*) in order to be able to transport water against gravity. Under low soil moisture, this water potential decreases, which eventually leads to the formation of air bubbles (ie embolism) within the vessels (Sperry and Tyree 1988). This process can substantially reduce water transport from the root to the shoots and can even completely block the hydraulic pathway, leading to tree mortality.

Some species are better equipped to resist drought than others, with a higher xylem resistance to embolism, measured as a lower *P_50_* or *P_88_*, the water potential at which 50% or 88% of the hydraulic conductivity is lost, respectively (Urli et al. 2013). To prevent the formation or spread of embolism, trees cut off the transpiration stream by closing stomata as one of the first strategies, thereby temporarily halting declines in water potential. Thus, at the leaf level, other common predictors of mortality risk include the water potential at which leaf turgor is lost (*P_TLP_*) or the water potential at which 50% (P_gs50_) or 90% (P_gs90_) of stomata close (Brodribb et al. 2003). However, after stomatal closure, water loss can still occur via either residual cuticular (g_min_) or bark (g_bark_) conductance (Wang et al. 2024). This is why trees with a higher water storage capacitance (C) may be more resistant or resilient to drought events, as they can gradually release and provide water to the plant during drought stress (Preisler et al. 2022).

As many previous studies have shown, these hydraulic traits are often coordinated and sit along an iso-anisohydric gradient (Fu and Meinzer 2019). Although this theory has been challenged (Feng et al. 2019), it reflects plant water-use strategies whereby isohydric plants will quickly close stomata upon drought stress while anisohydric plants tend to maintain gas exchange at gradually decreasing water potentials. These strategies may also alter the length of the drought response phases: first an initial leaf dehydration leading to stomatal closure and turgor loss; and second, a phase of embolism formation associated with the disruption of photochemical processes and eventually driving leaf and plant death (Martin-StPaul et al. 2017; Blackman et al. 2019). Thus, understanding and quantifying the length of these phases (first, the time to stomatal closure *t_sc_*, and second, the time to critical hydraulic failure *t_crit_*) can help predict the timing of a species drought survival.

In a new publication in *Plant Physiology*, Waite et al. (2026) explore how hydraulic traits are related to mortality risk across 16 tree species grown under gradual soil dry-down conditions. The authors chose 8 temperate broad-leaf species: 2 species of birch (*Betula maximowicziana* and *pendula*), 2 species of beech (*Fagus orientalis* and *sylvatica*), 2 species of oak (*Quercus petreae* and *rubra*) and 2 species of lime (*Tilia cordata* and *tomentosa*), and 8 conifers: 3 species of fir (Abies *alba*, *grandis*, and *Pseudotsuga menziesii*), 2 species of larch (*Larix decidua* and *kaempferi*), 2 species of pine (*Pinus nigra* and *sylvestris*) and 1 spruce (*Picea abies*), all of which are important in silviculture. The dry-down was induced by withholding irrigation. All relevant hydraulic traits were measured (*P_50_*, *P_88_*, *P_TLP_*, P_gs50_, P_gs90_, g_min_, g_bark_, and C). Additionally, predawn water potential (P_pd_) and whole shoot saturated water content (SWC_shoot_) were measured.

Across all species, Waite et al. (2026) found clear correlations between hydraulic traits. Traits linked to the first dry-down phase (ie before stomatal closure), such as *P_TLP_* and P_gs90_, were significantly correlated. Similarly, and as expected, the point considered to be the onset of embolism (ie the water potential when 12% of the xylem conductivity is lost, *P_12_*) was also correlated with *P_TLP_*. Indeed, in many species, leaves will lose turgor aiming to prevent any embolism event to form, which can lead to a large decline in stomatal conductance (Rodriguez-Dominguez et al. 2016). Interestingly, g_min_ was not strongly associated with other hydraulic traits. Instead, P_gs90_ and *P_50_* were strongly correlated, indicating that some species might maintain gas exchanges under decreasing water potential despite inducing xylem embolism. Moreover, *P_88_*, usually considered the hydraulic “point-of-no-return,” was correlated with wood density, g_bark_, and capacitance. These results suggest that trees displaying higher resistance to drought-induced embolism may require higher C in order to sustain a higher g_bark_ during dry-down (Salomón et al. 2017; Chen et al. 2026).

As anticipated, there were major differences between broad-leaf and conifer species. For instance, C was an important parameter linked to broad-leaf species, highlighting a key difference in the xylem anatomy. Indeed, water storage capacity is associated with wood anatomy (Tyree and Yang 1990) with broad-leaf species having a higher fraction of parenchyma and water-filled spaces in fibers, thereby enabling a higher capacity for water storage. On the other hand, g_min_ and g_bark_ were overall much lower in conifers, suggesting a stronger regulation of water loss in these plants (Wang et al. 2024).

Additionally, the authors modeled both *t_sc_* and *t_crit_* using a previously developed model from Blackman et al. (2019). The authors further improved the model by including species-specific responses and adding g_bark_. From this model, the authors were able to show that most of drought survival time happens before stomatal closure. Indeed, *t_sc_* accounted for 85% to 97% of the total survival time across species, indicating that this first phase was an important bottleneck. Surprisingly, this first phase, *t_sc_*, mainly correlated to xylem “safety” traits (ie *P_50_* and *P_88_*) but not stomatal traits. The second phase, *t_crit_*, was much shorter across all species and was mainly controlled by C, residual water loss both from leaves and bark, and was not regulated by embolism resistance. *t_crit_* was also much shorter in broad-leaf species, indicating that these species had shorter survival time after stomatal closure. Supported by previous studies (Copie et al. 2025), these results emphasize the importance of integrating a range of hydraulic traits instead of relying on single proxies to predict *t_sc_*.

Overall, the authors highlighted that tree drought survival might be better understood as a time-based, two-phase process (Fig. 1). Trait coordination was key to predicting survival outcomes and both phases were determined by different processes: while *t_sc_* was mainly correlated with xylem safety traits, *t_crit_* was determined by water storage and residual water loss. Finally, while broad-leaf and conifer species responded contrastingly to gradual dry-down, *t_sc_* was found to be the most critical determinant of drought sensitivity across all species. These results further underline the importance of moving beyond single metrics to predict drought vulnerability, instead using integrated and dynamic models. This could be used for forest management and silviculture recommendations in a changing world.

**Figure 1:**
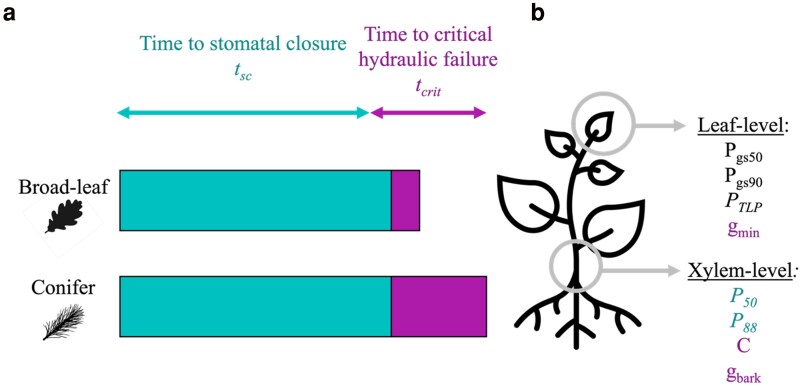
Critical metrics for predicting tree mortality. (a) Schematic representing the difference in time to stomatal closure (t_sc_, blue) and time to critical hydraulic failure (t_crit_, purple) in broad-leaf (top) and conifer (bottom) species. (b) List of traits associated with leaf (top) and xylem (bottom) hydraulics. Colored traits indicate their contribution to each phase: blue denotes traits contributing to t_sc_, while purple denotes traits contributing to t_crit_. Overall, time plays an important role to predict tree mortality and traits related to critical hydraulic failure are mostly related to water loss and capacitance.

## Data Availability

Data can be found in the highlighted article and its online supplementary material.
